# Multifocal clonal evolution characterized using circulating tumour DNA in a case of metastatic breast cancer

**DOI:** 10.1038/ncomms9760

**Published:** 2015-11-04

**Authors:** Muhammed Murtaza, Sarah-Jane Dawson, Katherine Pogrebniak, Oscar M. Rueda, Elena Provenzano, John Grant, Suet-Feung Chin, Dana W. Y. Tsui, Francesco Marass, Davina Gale, H. Raza Ali, Pankti Shah, Tania Contente-Cuomo, Hossein Farahani, Karey Shumansky, Zoya Kingsbury, Sean Humphray, David Bentley, Sohrab P. Shah, Matthew Wallis, Nitzan Rosenfeld, Carlos Caldas

**Affiliations:** 1 Cancer Research UK Cambridge Institute, University of Cambridge, Cambridge CB2 0RE, UK; 2 Department of Oncology, University of Cambridge, Cambridge CB2 0RE, UK; 3 Center for Noninvasive Diagnostics, Translational Genomics Research Institute, Phoenix, Arizona 85004, USA; 4 Mayo Clinic Center for Individualized Medicine, Scottsdale, Arizona 85259, USA; 5 Cambridge Breast Unit, Addenbrooke's Hospital, Cambridge University Hospital National Health Service Foundation Trust and National Institute for Health Research Cambridge Biomedical Research Centre, and the Cambridge Experimental Cancer Medicine Centre, Cambridge CB2 0QQ, UK; 6 Peter MacCallum Cancer Centre, East Melbourne, Victoria 3002, Australia; 7 Department of Histopathology, Addenbrooke's Hospital, Cambridge University Hospital NHS Foundation Trust, Cambridge CB2 0QQ, UK; 8 BC Cancer Research Centre, Vancouver, British Columbia, Canada V5Z 1L3; 9 Illumina, Inc., Chesterford Research Park, Little Chesterford CB10 1XL, UK; 10 Department of Radiology, Addenbrooke's Hospital, Cambridge University Hospital NHS Foundation Trust, Cambridge CB2 0QQ, UK

## Abstract

Circulating tumour DNA analysis can be used to track tumour burden and analyse cancer genomes non-invasively but the extent to which it represents metastatic heterogeneity is unknown. Here we follow a patient with metastatic ER-positive and HER2-positive breast cancer receiving two lines of targeted therapy over 3 years. We characterize genomic architecture and infer clonal evolution in eight tumour biopsies and nine plasma samples collected over 1,193 days of clinical follow-up using exome and targeted amplicon sequencing. Mutation levels in the plasma samples reflect the clonal hierarchy inferred from sequencing of tumour biopsies. Serial changes in circulating levels of sub-clonal private mutations correlate with different treatment responses between metastatic sites. This comparison of biopsy and plasma samples in a single patient with metastatic breast cancer shows that circulating tumour DNA can allow real-time sampling of multifocal clonal evolution.

## Abstract (web summary)

Individual tumours are heterogeneous with regards to genetic mutations. In this study, the authors use sequencing to follow multiple tumour and plasma samples over 3 years from a breast cancer patient and show mutations detected in the plasma samples could partially reproduce the clonal nature of the primary tumour.

## Graphical abstract

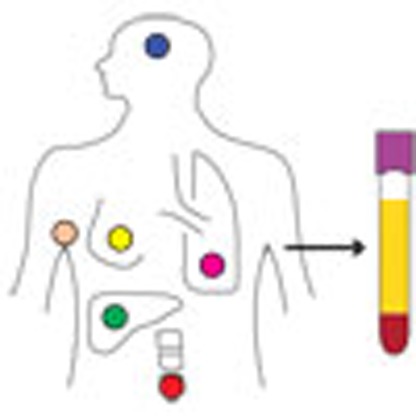

Intra-tumour clonal heterogeneity limits efficacy and duration of response to targeted treatments in metastatic cancer^1^,^2^,^3^. Evaluating heterogeneity to guide choice and sequence of therapy could be achieved by multiregional and repeated metastatic tumour biopsies but this is impractical due to associated risk of complications and costs. In contrast, analysis of circulating tumour DNA in plasma (ctDNA) is a less-invasive approach that could provide a summary of somatic alterations contributed by distinct metastases^4^,^5^, potentially circumventing the problem of spatial heterogeneity^1^. Serial analysis of ctDNA has been shown to track tumour burden^6^,^7^,^8^ and to correlate with treatment-driven clonal evolution^3^,^4^,^9^. Most studies of concordance between tumour and plasma samples have compared individual mutations or relied on single tumour biopsies^9^. However, direct evidence comparing plasma with multiregional tumour samples to establish the extent of clonal heterogeneity captured in ctDNA is extremely limited^5^,^10^,^11^,^12^,^13^.

Here we present extensive analysis of eight tumour biopsies and nine plasma samples collected from a patient with oestrogen receptor-positive (ER+) human epidermal growth factor receptor 2-positive (HER2+) metastatic breast cancer treated with sequential targeted therapies (tamoxifen and trastuzumab, followed by lapatinib) over a 3-year clinical course. We performed whole-exome followed by deep amplicon sequencing to validate and quantify several hundred somatic mutations. We find that ubiquitous stem mutations (common to all tumour biopsies) have the highest circulating levels in plasma followed by metastatic-clade and private mutations. In addition, serial changes during treatment in circulating levels of private somatic mutations correlate with disease progression in their respective tumour lesions on imaging. These results, from a single patient with metastatic breast cancer, suggest that ctDNA reflects clonal tumour hierarchy and captures sub-clonal dynamics in real time.

## Results

### Clinical case

A 42-year-old woman presented with a right breast lump, lower back pain, loss of height, marked kyphosis and hepatomegaly. Core biopsies from the breast lump showed ductal carcinoma *in situ* (sample labelled P1.1; Supplementary Fig. 1 and Supplementary Table 1). An additional biopsy from an ipsilateral axillary lymph node (P1.2) revealed metastatic ductal adenocarcinoma (ER+ (8/8) and HER2+ (3+)). Computed tomography scan revealed widespread metastatic disease in bones, pleura and liver (Supplementary Fig. 2 and Supplementary Table 2). The patient was started on treatment with trastuzumab and taxane-based chemotherapy, with a significant partial response (Supplementary Fig. 3). After induction chemotherapy, she was maintained on tamoxifen and trastuzumab. After 19 months on treatment, she presented with seizures and head computed tomography revealed a large metastasis in the left frontal lobe (Supplementary Fig. 4), which was resected (M2.1). Therapy with tamoxifen and trastuzumab was continued and collection of plasma samples was initiated (samples T1–T9). Four months after surgery, she had enlarging liver lesions and a new metastatic deposit in the left ovary (Supplementary Fig. 5). Treatment was switched to a combination of lapatinib and capecitabine, resulting in stable disease for 12 months (Supplementary Fig. 6). General deterioration then occurred, with disease progression in the chest (new pulmonary nodules, bilateral pleural effusions and posterior chest wall mass, Supplementary Fig. 7; Eastern Cooperative Oncology Group performance status 2–3). Treatment was stopped and the patient died ∼4 months later.

**Figure 1:**
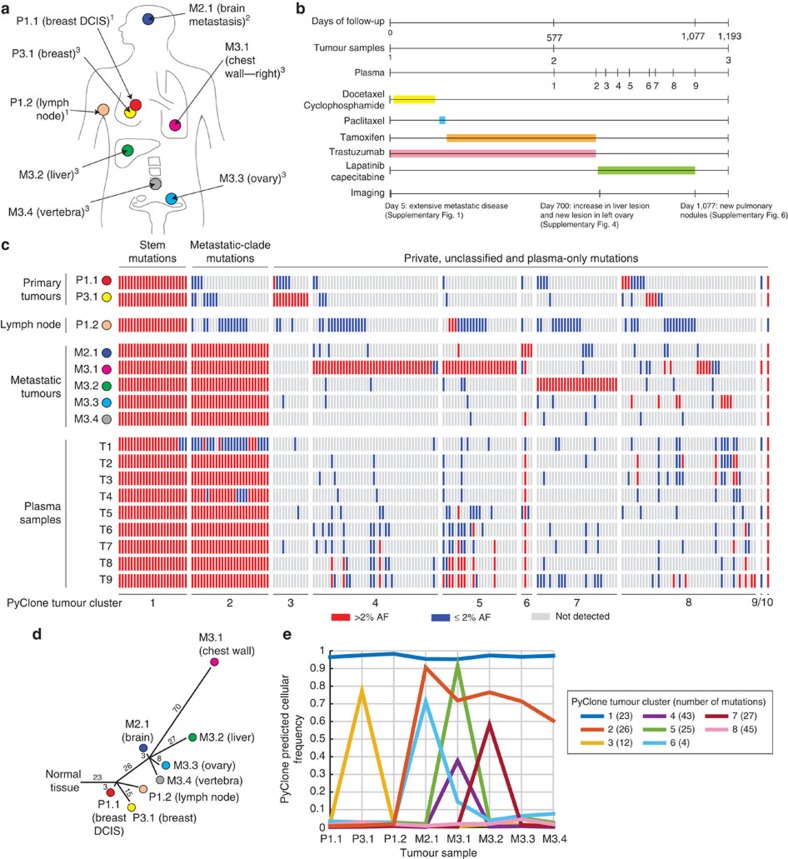
Inference of clonal structure from multiregional tumour biopsies. (**a**) Tumour samples collected from the patient, labelled P (primary) and M (metastasis). Numbers preceding dot (1,2 and 3) correspond to time of collection: 1, collected at diagnosis; 2, collected at the time of resection of brain metastasis; 3, collected at autopsy. (**b**) Timeline describing clinical course, samples collected, treatments administered and selected imaging assessments. Plasma DNA samples are labelled 1 through 9. Imaging assessments were performed using computed tomography scans. Histopathological and imaging findings are summarized in Supplementary Tables 1 and 2 and Supplementary Figs 1–6. (**c**) Distribution of 207 validated functional mutations in tumour and plasma samples, ordered by mutation clusters inferred using PyClone from mutant allele fractions in all tumours. Red rectangles indicate high-confidence mutations with AF >2%. Blue rectangles indicated mutations detected significantly above background but with AF of 2% or lower. Stem mutations (observed ubiquitously in all tumour samples and comprising tumour cluster 1) and metastatic-clade mutations (high confidence in metastatic tumours and comprising tumour cluster 2) are readily identifiable/detectable in plasma samples. Detailed values of allele fractions are documented in Supplementary Data 1–3. (**d**) Tumour phylogenetic tree, inferred by clonal ordering given distribution of high-confidence mutations in tumour samples shown in **a**. Length of each branch of the tree correlates with the number of mutations on the branch as indicated. Exome-sequencing results for samples P1.2 and M3.4 were not available and therefore private mutations for these branches cannot be identified. Assignment of mutations to each branch is documented in Supplementary Data 1. (**e**) Mean predicted cellular frequency of each cluster identified by PyClone across the tumour samples.

Tumour samples were obtained at diagnosis from the primary breast site (P1.1) and an axillary lymph node (P1.2); after 19 months from the brain metastasis area (M2.1); and at autopsy after 3 years on treatment (from the primary breast site, and from metastatic deposits in the chest, liver, ovary and vertebrae, labelled P3.1 and M3.1–M3.4, respectively). Serial plasma samples were obtained over the last 500 days of clinical follow-up (T1–T9). Tumour and plasma samples collected and the clinical course are summarized in Fig. 1a,b.

### Inferring clonal structure from multiregional tumour biopsies

Exome sequencing of peripheral blood leukocytes (N1), 6/8 tumour samples and 3/9 plasma DNA samples (3 plasma exomes reported previously^4^) was performed. Single-nucleotide variants (SNVs) were further analysed by targeted amplicon deep sequencing in all samples for orthogonal validation and accurate measurement of allele fractions (AFs, Supplementary Table 3). Of the 362 candidate non-synonymous SNVs identified by exome sequencing in at least one sample, 310 were successfully tested by deep sequencing (median coverage: 288 × –8,248 × for plasma samples; 965 × –2,777 × for tumour samples). For each candidate SNV, a mutation was called if AF was at least three s.d.'s above the mean background error rate obtained by analysing 12 control samples^11^.

Deep sequencing validated 207 functional mutations. We identified 8 major mutation clusters based on variation in their allele fractions across all tumour samples using Bayesian clustering with PyClone (Fig. 1c–e), a data-driven method we have developed and extensively validated for analysing clonal hierarchies and inferring cellular prevalence in tumour biopsies and to follow clonal dynamics in serially transplanted tumour xenografts^14^,^15^,^16^. We also inferred tumour phylogeny using clonal ordering of high-confidence mutations (with >2% allele fraction in a tumour sample). A total of 23 stem mutations were detected in all tumour samples (tumour cluster 1), 26 metastatic-clade mutations were detected only in metastatic tumour samples (tumour cluster 2) and 126 private mutations were detected at AF >2% only in one of the tumour samples (tumour clusters 3–8). The most parsimonious pathway of evolution in this cancer together with mutation clustering results is presented in Fig. 1d. Stem and metastatic-clade mutation clusters inferred using PyClone were identical to the results from clonal ordering. Similarly, mutations in clusters 3, 4/5, 6 and 7 correspond to private mutations in P3.1, M3.1, M2.1 and M3.2, respectively. A total of 13/26 metastatic-clade mutations were detectable at low levels in the lymph node biopsy samples (P1.2), consistent with a common ancestor for metastasis as a minor clone at the axillary lymph node site. The inferred phylogenetic structure was stable using 5 and 10% allele fraction cutoffs for high-confidence mutations (Supplementary Figs 8 and 9) and allele fractions for stem mutations were highly correlated between all tumour samples (Supplementary Fig. 10).

### Serial plasma analysis and comparison with tumours

**Figure 2:**
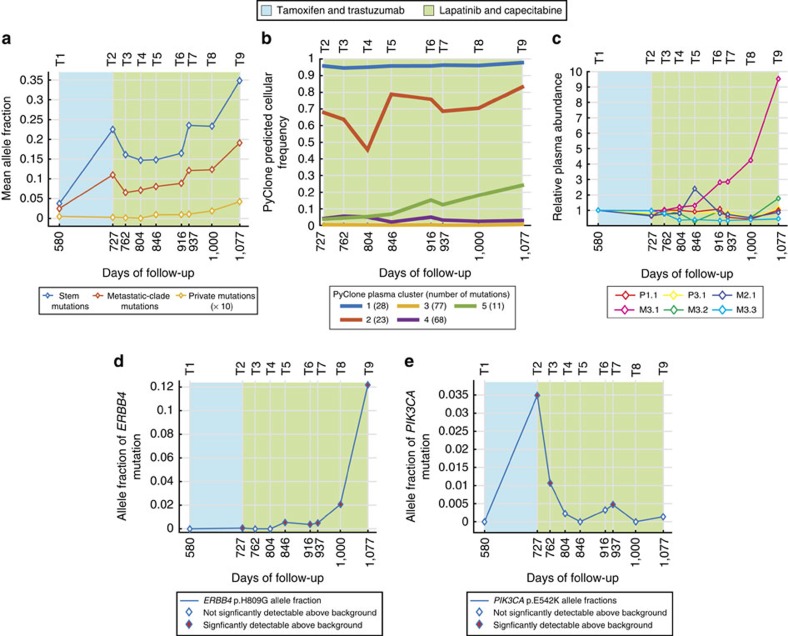
Serial plasma analysis during systemic treatment. (**a**) Circulating levels of stem, metastatic-clade and private mutations during treatment. Mean allele fractions at each time point are presented. Mean AF for private mutations is multiplied by 10 to highlight trend. Shaded areas represent treatment lines. (**b**) Mean predicted cellular frequency of each cluster identified by PyClone across the plasma samples T2–T9. PyClone identified five mutation clusters from variation of circulating allele fractions (without reliance on tumour data). Clusters 1 and 2 are largely comprised of stem and metastatic-clade mutations. Cluster 5 is comprised of 11 mutations, 10 of which are private M3.1 mutations. (**c**) Plasma abundance calculated as the product of AF in a tumour sample (normalized for mean of stem mutations) and the corresponding AF in a plasma sample, summed across all private mutations for each tumour. To normalize for different number of private mutations in each tumour (3–70), we calculated plasma abundance relative to T1. (**d**) Dynamics of *ERBB4* mutation (p.H809G) in deep sequencing data. (**e**) Allele fractions measured by deep amplicon sequencing for the *PIK3CA* mutation (p.E542K) identified in exome sequencing of plasma sample T2. Mutation was significantly detectable (>3 s.d.'s above the mean allele fraction in control samples) on days 727, 762 and 937 (yellow diamonds).

In plasma, stem mutations were highest in abundance, with mean plasma AFs ranging from 3.8 to 34.9% across the time series. Metastatic-clade mutations were lower in abundance with mean AFs ranging from 2.5 to 19.1% (Wilcoxon rank sum test *P*<0.001, except T5 *P*=0.001). The dynamic longitudinal changes in plasma AFs for both mutation groups reflected the observed overall tumour response, both clinically and on imaging (Fig. 2a). Mutation clusters statistically inferred using PyClone from variation in circulating mutant allele fractions (without relying on tumour data, referred to as ‘plasma clusters') overlapped significantly with clusters identified from multiregional tumour sampling. A total of 21/23 stem mutations were assigned to plasma cluster 1 (with highest cellular prevalence), and 19/26 metastatic-clade mutations were assigned to plasma cluster 2 (Fig. 2b and Supplementary Data 1).

To assess whether plasma DNA captured differential response across distinct metastatic sites during targeted treatment, the relative plasma abundance of high-confidence private mutations originating from each tumour site was calculated. During lapatinib treatment, a rapid increase in the circulating abundance of several mutations private to the chest mass was observed in plasma samples T4–T9 (Fig. 2c), coinciding with significant disease progression seen on imaging at this site. This was also reflected in plasma-based PyClone mutation clusters; plasma cluster 5 increased in circulating prevalence with disease progression on lapatinib treatment and 10/11 mutations in this cluster are private to M3.1 (and correspond to tumour clusters 4 and 5; Fig. 2b and Supplementary Data 1). At the time of lapatinib resistance, the most abundant private mutation in plasma was in the tyrosine kinase domain of *ERBB4* (p.H809G; plasma cluster 5; Fig. 2b,d and Supplementary Fig. 11). This mutation was private to the chest wall mass (28.2% AF) with its levels in plasma DNA increasing during lapatinib treatment up to an AF of 12.2% at the time of disease progression on imaging (compared with average stem and metastatic-clade AFs of 34.9 and 19.1% in the same plasma sample). The predicted functional effect of this mutation^17^,^18^ and its exclusive molecular detection in the chest wall mass (the main site of disease progression on treatment) suggest it was a key determinant of resistance to lapatinib.

Interestingly, 11 non-synonymous high-confidence SNVs were identified and validated in plasma but not detectable at >2% AF in any of the analysed tumour biopsies. Amongst these was an actionable hotspot mutation in *PIK3CA* (p.E542K), identified in plasma with an AF of 3.5% at the time of progression on trastuzumab and tamoxifen (tumour cluster 8 and plasma cluster 4; Fig. 2e). After lapatinib treatment was started, the plasma levels dropped to AF of 1.1% and then became undetectable. This mutation was only marginally detectable (AF <1%) in two tumour biopsies (axillary lymph node and vertebral metastasis). These results suggest this *PIK3CA* mutation originated from a minor tumour sub-clone that increased in size during treatment with tamoxifen and trastuzumab, and then regressed on treatment with lapatinib. Activation of the PI3K/AKT pathway has been associated with resistance to both endocrine therapy and trastuzumab^19^,^20^.

## Discussion

In this paper, we have presented an extensive comparison of biopsy and plasma samples collected from a metastatic breast cancer patient over a 3-year clinical course. Our results show that circulating tumour DNA provides a dynamic sampling of somatic alterations reflecting the size and activity of distinct tumour sub-clones. Analysis of ctDNA reflects the clonal hierarchy determined from multiregional tumour sequencing and tracks different treatment responses across metastases. Unlike previous reports, our results qualify tumour-plasma concordance of each somatic mutation in context of the tumour phylogeny. Truncal mutations that represent the majority of tumour lesions in a patient have higher circulating levels and therefore, are more likely to be detected in plasma, than clade or private mutations.

These results were obtained from deep analysis of a single patient and need to be confirmed in a larger cohort of patients with multiregional biopsies and serial plasma samples. If confirmed, our observations have important implications for future ctDNA studies. For monitoring tumour burden using ctDNA, our results suggest that truncal mutations are the best candidates, as they are highest in circulating levels and least likely to drop out during follow-up. For molecular treatment stratification, our results suggest that if multiple actionable somatic mutations, or alterations that are known to confer resistance to specific therapies, are identified in tumour analysis, their relative circulating levels in pretreatment plasma samples may inform the choice of targeted treatments for individual patients. The potential of using plasma DNA for molecular stratification and tracking of resistant clones in patients treated with targeted therapies heralds a new era for precision cancer medicine.

## Methods

### Sample collection and exome sequencing

Informed consent was obtained and research autopsy was performed under a study protocol approved by the Cambridgeshire Research Ethics Committee (Cambridgeshire 3 REC 07/Q0106/63MN.A). Collection, processing, DNA extraction and preparation of exome-sequencing libraries for plasma samples T1, T2 and T9 have been described previously^4^. Exome sequencing of tumour samples and additional sequencing of germline DNA (N1) was performed using commercially available kits. Tumour and germline DNA were sheared using sonication to a target fragment size of 200 bp. Whole-genome libraries were prepared from 32 to 50 ng of fragmented DNA using ThruPLEX-FD (Rubicon Genomics) as per the manufacturer's protocols, with unique sample-specific molecular barcodes. Genomic libraries were quantified using quantitative PCR and pooled for exome enrichment by hybridization using the TruSeq Exome Enrichment Kit (Illumina). Enriched libraries were quantified using quantitative PCR and pooled for sequencing on the HiSeq 2500 (Illumina).

### Targeted amplicon sequencing

Targeted sequencing libraries were prepared using droplet-based PCR amplification following the manufacturer's protocols for ThunderBolts Cancer Panel with specific modifications (RainDance Technologies). Custom target-specific primers were designed using in-house primer design pipelines (see Supplementary Data 5 for the list of primer sequences). Universal adapters were added on the 5′-end to allow sample-specific barcoding. Target-specific amplification was performed using primers flanking 350 loci in multiplex in a 40-μl volume PCR mix. Primer concentration was limited to 3.5 nM per primer (an estimated 10,000 copies per 5 pl droplet). Droplets were generated on the RainDrop Source instrument (8,000,000 droplets for a 40-μl volume). An input of 2–18 ng (mean: 12.1 ng) of plasma DNA (1–10-μl volume of eluted DNA), corresponding to the DNA extracted from a volume of 40–400 μl (mean: 280 μl) of plasma, and 6–31 ng (mean: 21.5 ng) of genomic DNA from tumour and germline samples were used for library preparation. PCR was performed for 55 cycles using 1 °C s^−1^ ramp and following conditions: 94 °C for 30 s, 62 °C for 30 s and 68 °C for 1 min followed by a final extension at 68 °C for 10 min. Droplets were destabilized using manufacturer-supplied reagents. PCR product was purified using magnetic beads (SPRIworks) in 2:1 volume ratio. PCR product was eluted in 20 μl 1 × Tris–EDTA buffer (pH 8.0). A second 25 μl barcoding PCR was performed using 13 μl of the eluted product and primers specific to the universal adapter with sample-specific barcodes. PCR was performed for 10 cycles using 1 °C s^−1^ ramp and following conditions: 94 °C for 30 s, 56 °C for 30 s and 68 °C for 1 min followed by a final extension at 68 °C for 10 min. An additional purification was performed using magnetic beads (SPRIworks) in a 1.2:1 volume ratio. Libraries were quantified using KAPA SYBR FAST LightCycler 480 qPCR kit (KAPA Biosystems) and using DNA High Sensitivity Kit on BioAnalyzer (Agilent Technologies) and pooled in 1:1 ratio. Paired-end sequencing was performed using MiSeq 150-cycle v3 kit (Illumina).

### Exome-sequencing analysis and mutation calling

Sequencing reads were demultiplexed allowing zero mismatches in barcodes. Paired-end alignment to the hg19 genome was performed using BWA version 0.5.9 for all exome-sequencing data including germline samples, plasma samples and tumour samples^21^. PCR duplicates were marked using Picard. Local realignment was performed using Genome Analysis Tool Kit^22^. Pileup files were generated for the genomic regions targeted by exome enrichment using samtools v0.1.1722 (ref. 23). For plasma samples, properly paired reads with mapping quality ≥60 were used to generate the pileup. AFs for each single-base locus were calculated for all bases with phred quality ≥30. For germline DNA, an additional pileup file was generated (using a mapping quality cutoff of ≥1 and without any base quality cutoffs) and was used as reference for calling somatic variants. All mutations were annotated for genes and function as well as repeated genomic regions using ANNOVAR^24^.

A mutation was identified if (1) no mutant reads for an allele were observed in germline DNA (N1) at a locus that was covered at least 10-fold, (2) at least five reads supporting the mutant were observed in any tumour or plasma sample with at least one read on each strand (forward and reverse) and (3) the binomial probability of observing the number of mutant reads given total depth at that locus was <0.001 assuming an error rate of 0.01.

### Analysis of targeted sequencing data

Sequencing reads were extracted and demultiplexed using Picard allowing zero mismatches in barcodes and a base quality of ≥30. Sequencing reads were clipped to remove universal adapter sequences using ea-utils. Minimum amplicon length in our set was 80 bp. Therefore, we removed any sequencing reads <70 bp in length following adapter clipping to discard nonspecific amplification and primer dimers. Clipped sequencing reads were aligned to the human genome hg19 using BWA version 0.7.10. Unmapped reads, unpaired reads and supplementary alignments were removed. As described previously, reads were demultiplexed to specific amplicons using known amplicon start and end positions and expected amplicon length (accounting for potential indels)^11^. Pileup files were generated using samtools including any reads with mapping quality ≥30 and base quality ≥30. Pileup data were imported into MATLAB.

For each locus and non-reference allele of interest, we assessed the allele fraction in eight control plasma samples and four control genomic DNA samples. We considered a mutation significantly detectable if the AF in a sample was >3 s.d.'s higher than the mean AF in control samples.

### Control samples

A volume of 250 ml pooled control plasma sample was purchased from BioreclamationIVT (Baltimore, MD, USA). The sample was prepared from equal number of male and female volunteers and collected with K2 EDTA additive. We performed independent cell-free DNA extractions from 1-ml aliquots of plasma and eight aliquots were used as control plasma samples. Four genomic DNA control samples were used from the Human Random Control DNA Panel 3 (Sigma-Aldrich).

### Calculation of plasma abundance for private mutations

Plasma abundance was calculated as the product of AF of a private mutation in a tumour sample and the corresponding AF in a plasma sample, summed across all private mutations for each tumour. To account for cellularity of each tumour sample, we normalized the tumour AF of each mutation by mean tumour AF of stem mutations. To normalize for different number of private mutations in each tumour, we calculated plasma abundance relative to T1.

### Bayesian clustering using PyClone

PyClone (a Bayesian clustering method) was used to infer the clonal population structures present in the tumour and plasma samples from the amplicon sequencing data. Given the mutation allele frequencies for each sample, PyClone clusters mutations that shift together across the samples and estimates cellular prevalence for each cluster in each sample (adjusting for copy number changes and normal cell contamination). To infer the clonal population structure of each sample (either tumour sample or plasma sample), copy number and depth of coverage information must be determined for each mutation under analysis.

Copy number information at each mutation location was generated from the whole-exome-sequencing data using the CopyWriteR Bioconductor package. CopyWriteR uses off-target read information from targeted sequencing data files to determine copy number. CopyWriteR outputs segmented logarithmic depth of coverage ratios (logR), which are converted to absolute copy number predictions. Segments with logR below −0.25 were assigned a copy number of 1 and those with logR above 0.25 received a copy number of 3. A bin size of 100 kb was utilized with hg19 as the reference genome. Whole-exome-sequencing data were available from four metastatic samples, two primary tumour samples, three plasma samples and patient's germline DNA sample (used as the control in copy number determination). Copy number predictions for other samples were assigned as the median copy number calculated for all available samples of the same type (either plasma, primary or metastasis). If the algorithm was unable to deduce copy number at a given mutation locus, the sequentially nearest valid copy number assignment was used. Inferred total copy number information for tumour and plasma samples is presented in Supplementary Data 4.

Depth of coverage (for the normal and variant alleles at each mutation) was computed using the bam2R function in the deepSNV Bioconductor package. The amplicon sequencing files for each sample were used as input. Reads with a phred quality of 30 or greater were included in the recorded read counts.

Depth of coverage and copy number information for each mutation was then inputted into PyClone (a Bayesian clustering method) to infer the presence of clonal mutations in both the tumour and plasma samples. Two PyClone analyses were performed: one for the tumour samples and another for the plasma samples. For each simulation, the PyClone algorithm was run for 40,000 iterations with a burn-in of 20,000 iterations using the PyClone beta binomial model with the ‘total_copy_number' option. A beta binomial value of 500 was utilized. Default values were used for all other parameters. Cellularity for each sample (including ctDNA samples) was estimated by computing the mean allele fraction for mutations classified as ‘stem mutations'—these are reported in Supplementary Table 1. Mean predicted cellular frequencies (in the case of ctDNA these should be interpreted as clonal frequencies) for each cluster identified by PyClone are plotted in the Figs 1e and 2b. Because PyClone corrects for normal cell contamination, the predicted cellular frequencies shown in the figures represent the proportion of cancer cells containing each set of clonal mutations (hence stem mutation cluster in plasma being near 100% frequency). The T1 plasma sample was not included in the PyClone analyses; data from the T1 sample were uncharacteristically noisy due to the sample's low cellularity (3%)—a reflection of low systemic tumour burden mid-treatment. The PyClone inference results for two mutations (in the tumour sample simulation) were ambiguous (the 5th–95th percentile credible range from the PyClone post-burn-in trace data spanned more than 70% of the cellular frequency space), leading to singleton clusters for each. Two mutations are not shown in Fig. 1e.

## Additional information

**Accession codes:** The sequencing data have been deposited at the European Genome-phenome Archive (EGA), which is hosted by the EBI, under accession code EGAS00001001466.

**How to cite this article:** Murtaza, M. *et al.* Multifocal clonal evolution characterized using circulating tumour DNA in a case of metastatic breast cancer. *Nat. Commun.* 6:8760 doi: 10.1038/ncomms9760 (2015).

## Supplementary Material

Supplementary Information Supplementary Figures 1-11, Supplementary Tables 1-3 and Supplementary Reference

Supplementary Data 1 Allele fractions of functional mutations validated.

Supplementary Data 2 Normalized abundance of all functional mutations validated.

Supplementary Data 3 Allele fractions and normalized abundance of validated mutations reported as enriched in ctDNA previously

Supplementary Data 4 Total copy number at mutant loci inferred from whole exome sequencing of tumor and plasma samples.

Supplementary Data 5 Custom primers used for targeted amplicon sequencing

## References

[b1] Aparicio S. & Caldas C. The implications of clonal genome evolution for cancer medicine. N. Engl. J. Med. 368, 842–851 (2013). 23445095 10.1056/NEJMra1204892

[b2] Gerlinger M. *et al.* Intratumor heterogeneity and branched evolution revealed by multiregion sequencing. N. Engl. J. Med. 366, 883–892 (2012). 22397650 10.1056/NEJMoa1113205 PMC4878653

[b3] Diaz L. A. Jr *et al.* The molecular evolution of acquired resistance to targeted EGFR blockade in colorectal cancers. Nature 486, 537–540 (2012). 22722843 10.1038/nature11219 PMC3436069

[b4] Murtaza M. *et al.* Non-invasive analysis of acquired resistance to cancer therapy by sequencing of plasma DNA. Nature 497, 108–112 (2013). 23563269 10.1038/nature12065

[b5] Chan K. C. *et al.* Cancer genome scanning in plasma: detection of tumour-associated copy number aberrations, single-nucleotide variants, and tumoral heterogeneity by massively parallel sequencing. Clin. Chem. 59, 211–224 (2013). 23065472 10.1373/clinchem.2012.196014

[b6] Dawson S. J. *et al.* Analysis of circulating tumour DNA to monitor metastatic breast cancer. N. Engl. J. Med. 368, 1199–1209 (2013). 23484797 10.1056/NEJMoa1213261

[b7] Diehl F. *et al.* Circulating mutant DNA to assess tumour dynamics. Nat. Med. 14, 985–990 (2008). 18670422 10.1038/nm.1789 PMC2820391

[b8] Newman A. M. *et al.* An ultrasensitive method for quantitating circulating tumour DNA with broad patient coverage. Nat. Med. 20, 548–554 (2014). 24705333 10.1038/nm.3519 PMC4016134

[b9] Bettegowda C. *et al.* Detection of circulating tumour DNA in early- and late-stage human malignancies. Sci. Transl. Med. 6, 224ra224 (2014). 10.1126/scitranslmed.3007094 PMC4017867 24553385

[b10] Bashashati A. *et al.* Distinct evolutionary trajectories of primary high-grade serous ovarian cancers revealed through spatial mutational profiling. J. Pathol. 231, 21–34 (2013). 23780408 10.1002/path.4230 PMC3864404

[b11] Forshew T. *et al.* Noninvasive identification and monitoring of cancer mutations by targeted deep sequencing of plasma DNA. Sci. Transl. Med. 4, 136ra168 (2012). 10.1126/scitranslmed.3003726 22649089

[b12] Heitzer E. *et al.* Tumor-associated copy number changes in the circulation of patients with prostate cancer identified through whole-genome sequencing. Genome Med. 5, 30 (2013). 23561577 10.1186/gm434 PMC3707016

[b13] De Mattos-Arruda L. *et al.* Capturing intra-tumour genetic heterogeneity by de novo mutation profiling of circulating cell-free tumour DNA: a proof-of-principle. Ann. Oncol. 25, 1729–1735 (2014). 25009010 10.1093/annonc/mdu239 PMC6276937

[b14] Eirew P. *et al.* Dynamics of genomic clones in breast cancer patient xenografts at single-cell resolution. Nature 518, 422–426 (2015). 25470049 10.1038/nature13952 PMC4864027

[b15] Roth A. *et al.* PyClone: statistical inference of clonal population structure in cancer. Nat. Methods 11, 396–398 (2014). 24633410 10.1038/nmeth.2883 PMC4864026

[b16] Shah S. P. *et al.* The clonal and mutational evolution spectrum of primary triple-negative breast cancers. Nature 486, 395–399 (2012). 22495314 10.1038/nature10933 PMC3863681

[b17] Qiu C. *et al.* Mechanism of activation and inhibition of the HER4/ErbB4 kinase. Structure 16, 460–467 (2008). 18334220 10.1016/j.str.2007.12.016 PMC2858219

[b18] Canfield K. *et al.* Receptor tyrosine kinase ERBB4 mediates acquired resistance to ERBB2 inhibitors in breast cancer cells. Cell Cycle 14, 648–655 (2015). 25590338 10.4161/15384101.2014.994966 PMC4614407

[b19] Berns K. *et al.* A functional genetic approach identifies the PI3K pathway as a major determinant of trastuzumab resistance in breast cancer. Cancer Cell 12, 395–402 (2007). 17936563 10.1016/j.ccr.2007.08.030

[b20] Chandarlapaty S. *et al.* Frequent mutational activation of the PI3K-AKT pathway in trastuzumab-resistant breast cancer. Clin. Cancer Res. 18, 6784–6791 (2012). 23092874 10.1158/1078-0432.CCR-12-1785 PMC3525734

[b21] Li H. & Durbin R. Fast and accurate short read alignment with Burrows-Wheeler transform. Bioinformatics 25, 1754–1760 (2009). 19451168 10.1093/bioinformatics/btp324 PMC2705234

[b22] DePristo M. A. *et al.* A framework for variation discovery and genotyping using next-generation DNA sequencing data. Nat. Genet. 43, 491–498 (2011). 21478889 10.1038/ng.806 PMC3083463

[b23] Li H. *et al.* The sequence alignment/map format and SAMtools. Bioinformatics 25, 2078–2079 (2009). 19505943 10.1093/bioinformatics/btp352 PMC2723002

[b24] Wang K., Li M. & Hakonarson H. ANNOVAR: functional annotation of genetic variants from high-throughput sequencing data. Nucleic Acids Res. 38, e164 (2010). 20601685 10.1093/nar/gkq603 PMC2938201

