# Supplementary material for: Multifocal clonal evolution characterized using circulating tumour DNA in a case of metastatic breast cancer
Source: Nat Commun. 2015 Nov 4;6:8760. doi: 10.1038/ncomms9760 (PMC4659935; doi:10.1038/ncomms9760)
Supplement: Supplementary Information — Supplementary Figures 1-11, Supplementary Tables 1-3 and Supplementary Reference [file ncomms9760-s1.pdf]

Supplementary Information

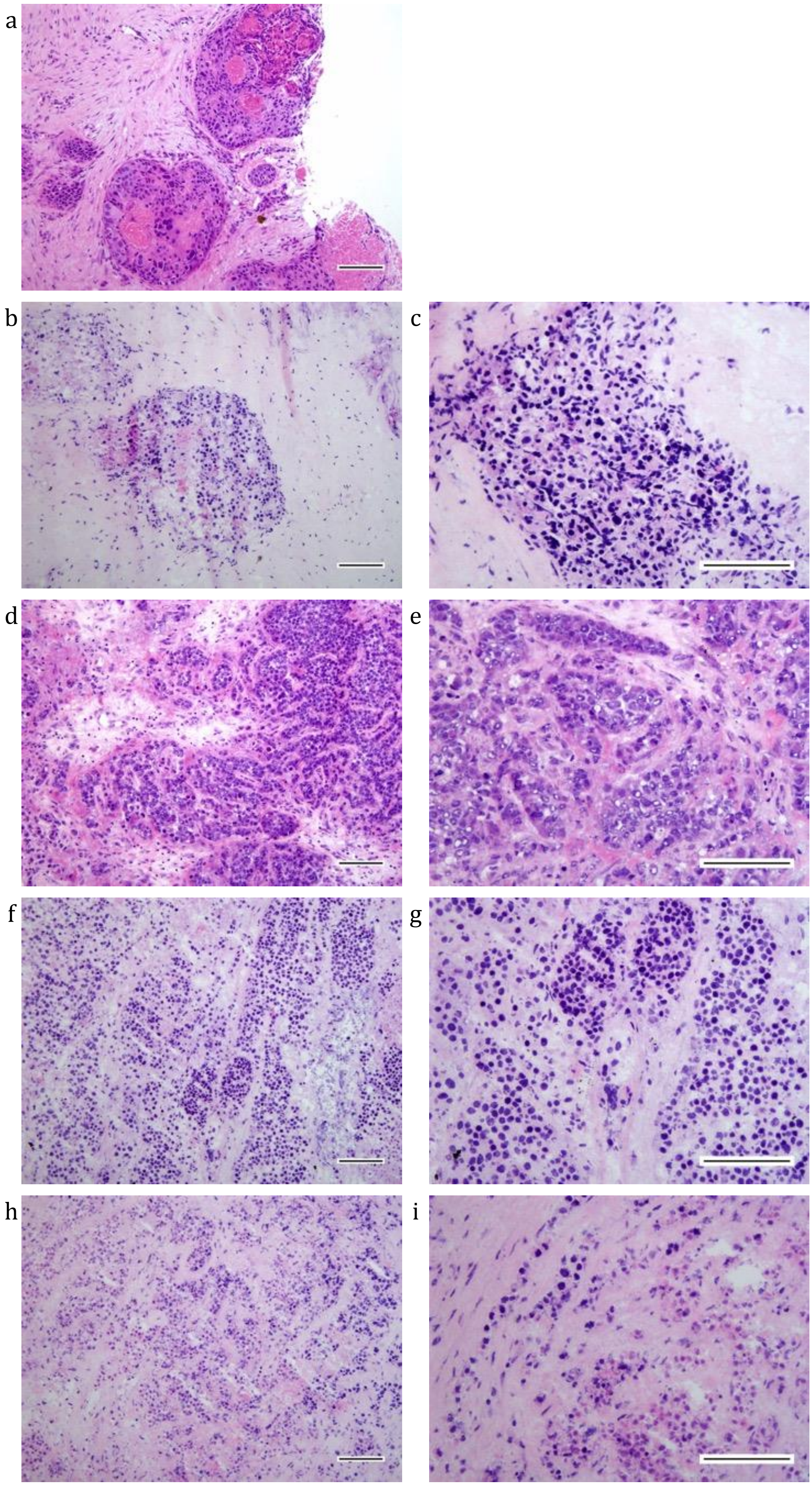

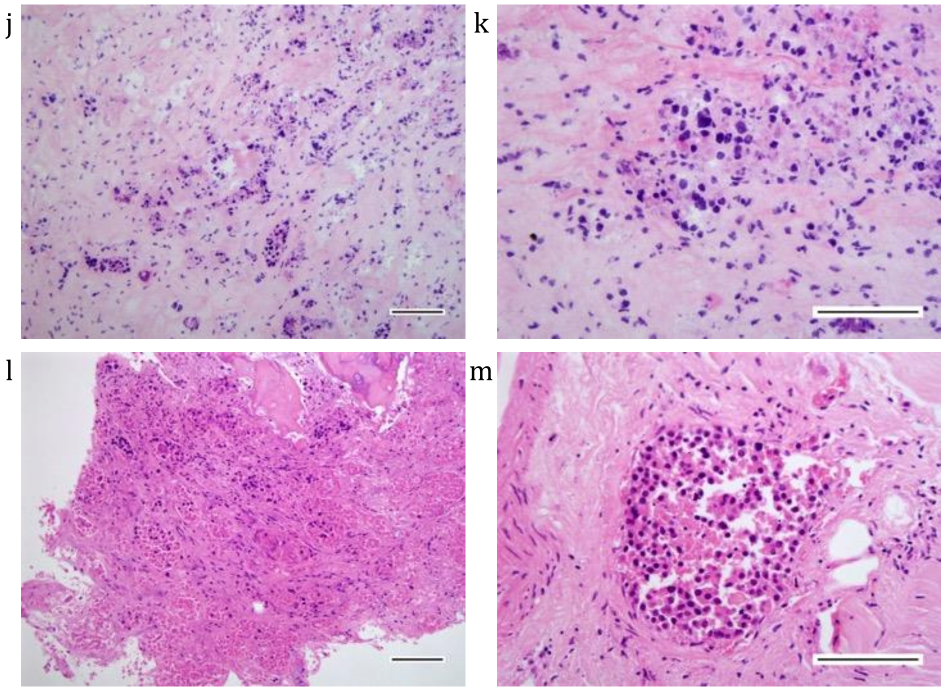

Supplementary Figure 1. Representative images from histopathology slides from tumor specimen. Images on the left are all at 10x magnification while those on the right are all at 20x magnification. Scale bars are 100 µm. (a) P1.1 (b,c) P3.1 (d,e) M2.1 (f,g) M3.1 (h,i) M3.2 (j,k) M3.3 (l,m) M3.4

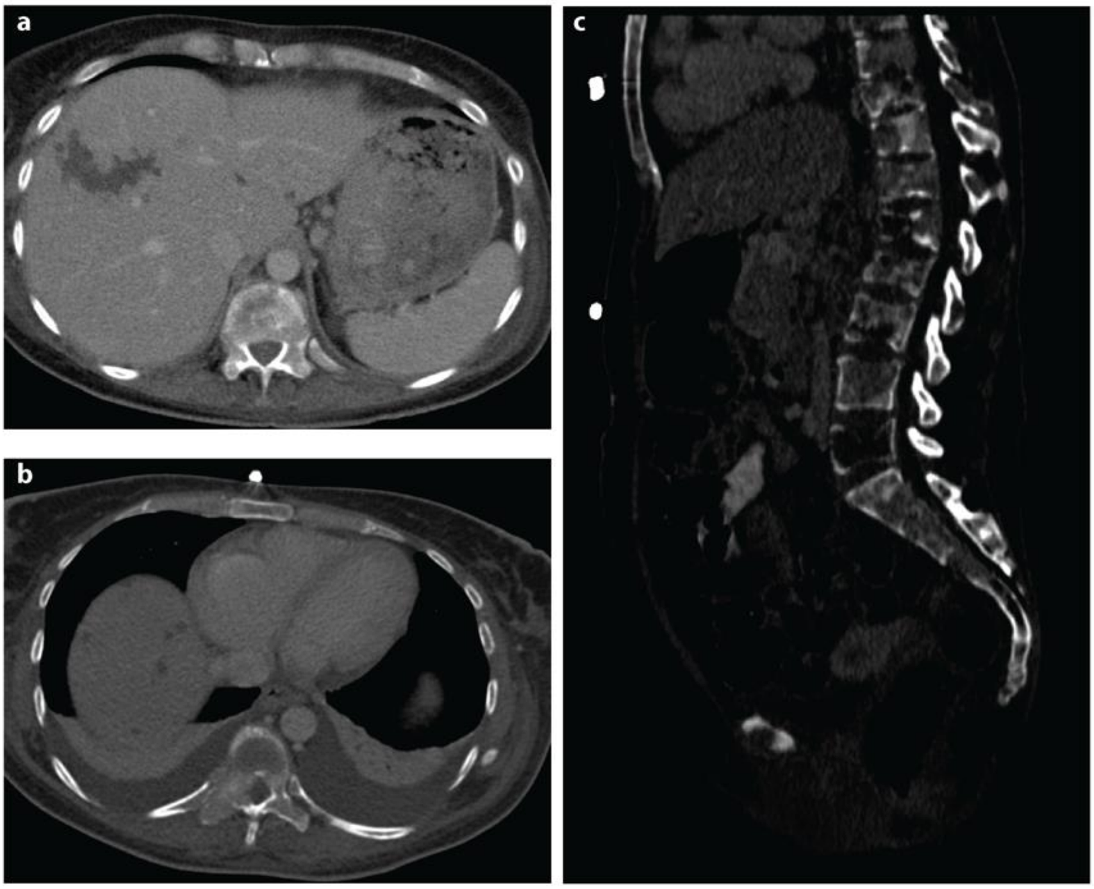

Supplementary Figure 2. Representative images from CT scan at diagnosis (day 5). (a) Largest confluent lesion in the liver. (b) Bilateral pleural effusion and basal atelectasis. (c) Sagittal view showing diffuse vertebral metastases.

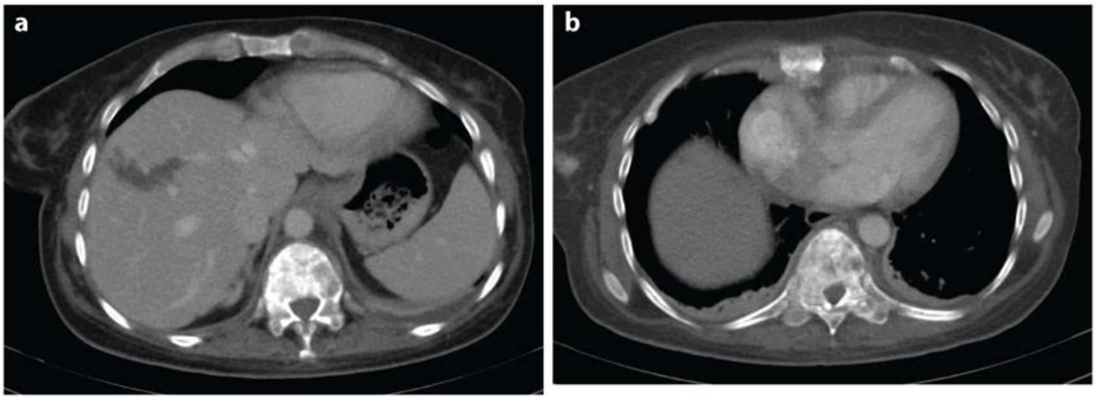

Supplementary Figure 3. Representative images from CT scan following partial response to trastuzumab (day 75). (a) Liver lesion, reduced in size. (b) Resolution of bilateral pleural effusion

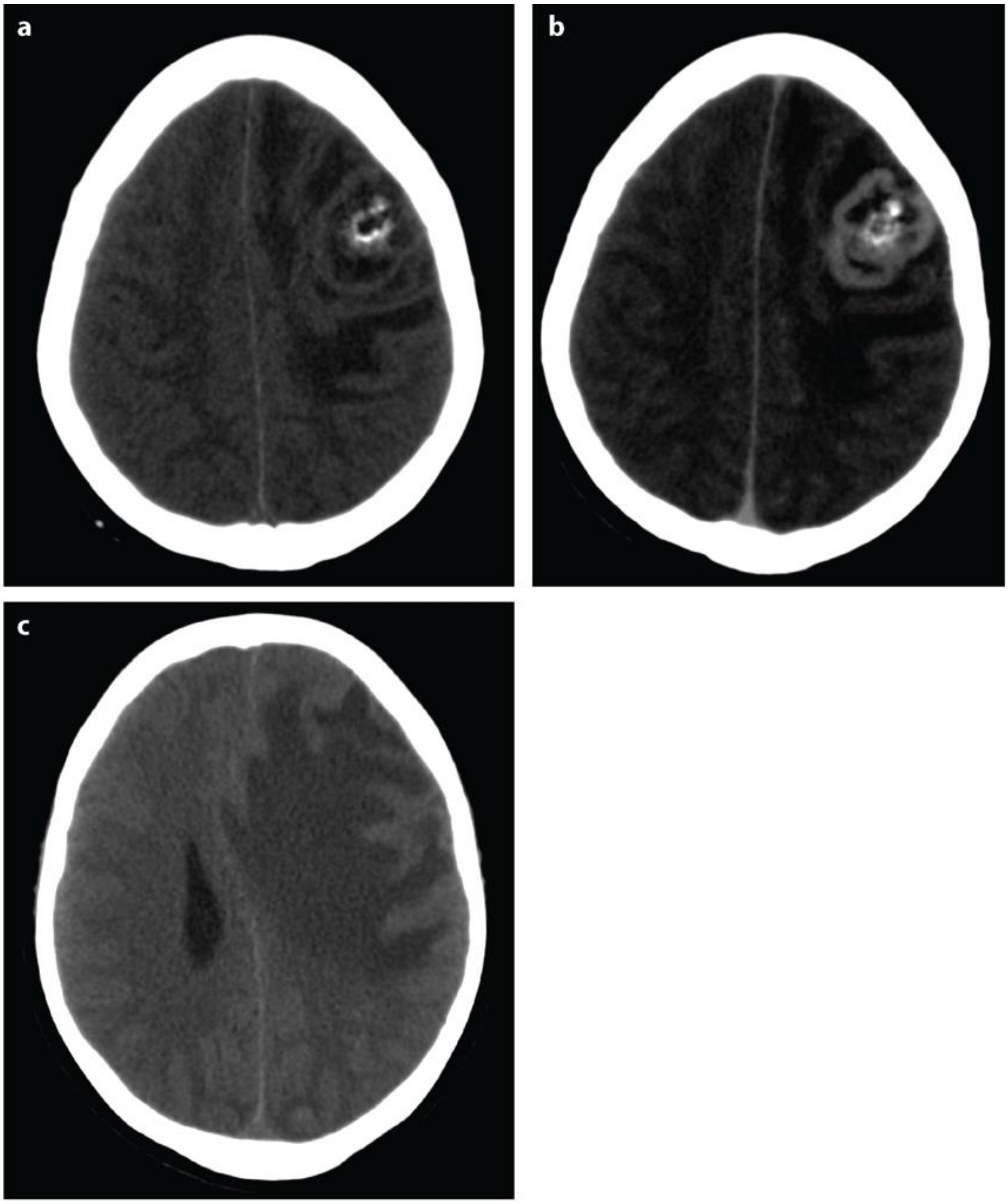

Supplementary Figure 4. Representative images from CT scan of the head performed when the patient presented with seizures (day 564). (a,b) Contrast enhancing lesion with central calcification seen in the left frontal lobe. (c) Surrounding edema and midline shift.

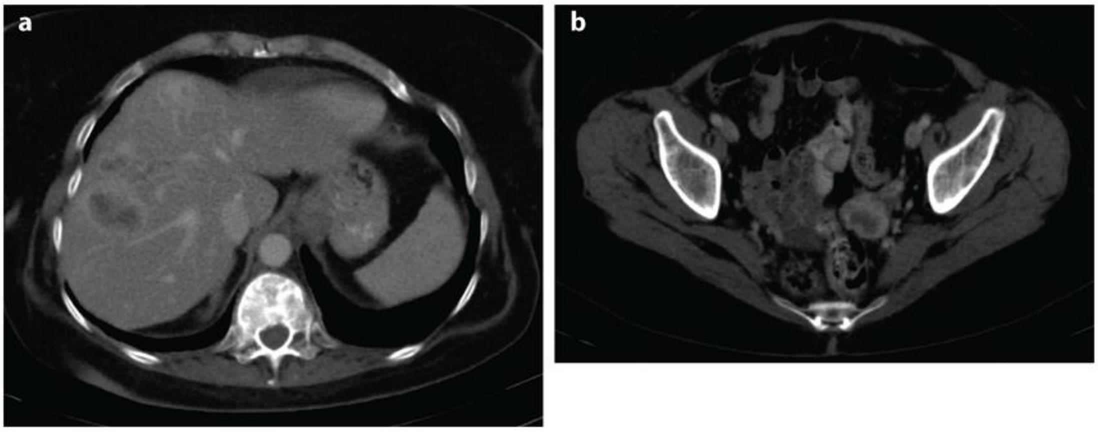

Supplementary Figure 5. Representative images from CT scan at the time of progression on trastuzumab and tamoxifen (day 700). (a) Liver lesion, increasing in size. (b) Newly observed left ovarian lesion.

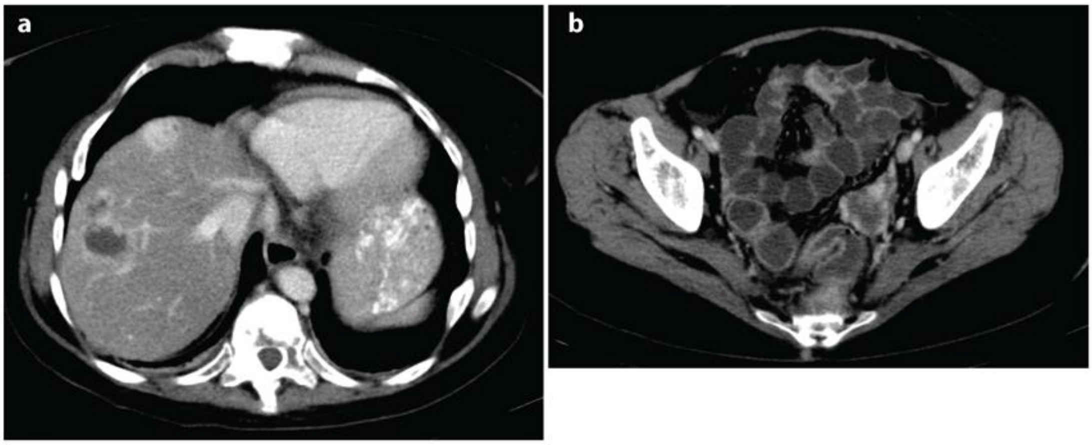

Supplementary Figure 6. Representative images from CT scan showing stable disease on treatment with lapatinib and capecitabine (day 804). (a) Liver lesion, slight decrease in size. (b) Left ovarian lesion, no change in size.

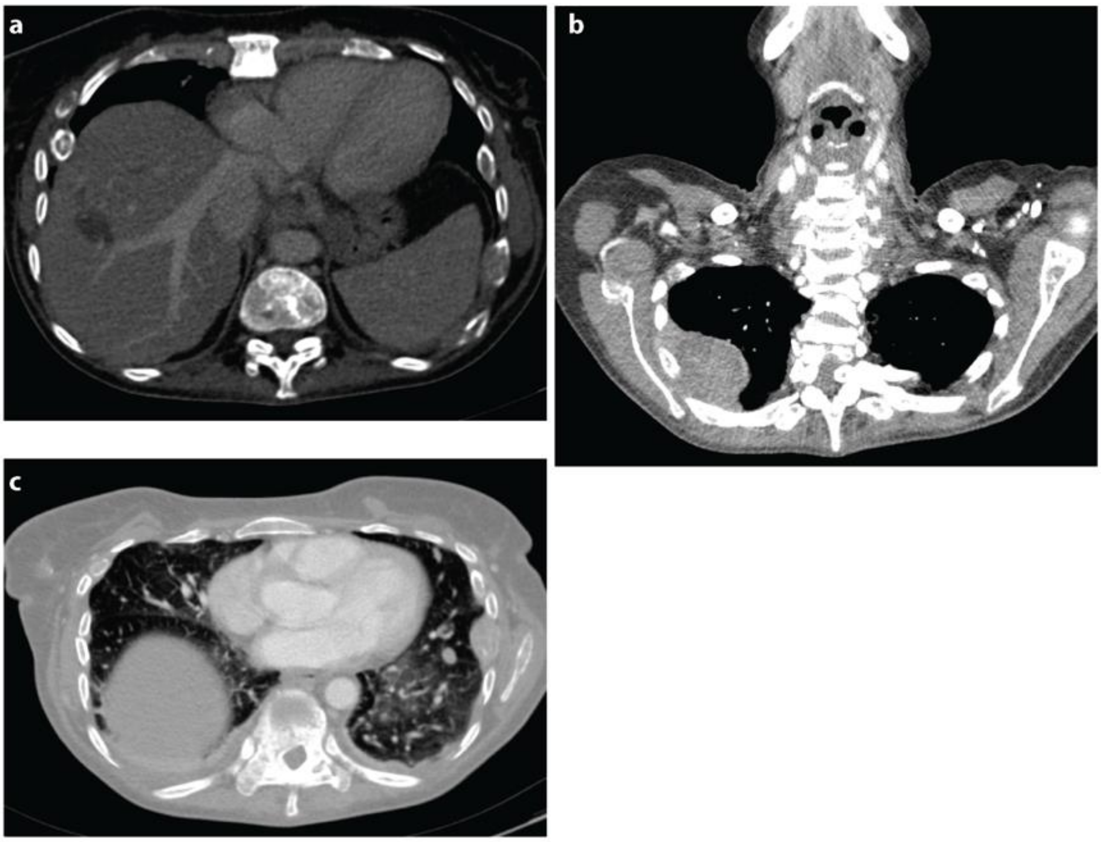

Supplementary Figure 7. Representative images from CT scan at the time of progression on treatment with lapatinib and capecitabine (day 1077). (a) Liver lesion. (b) Right posterior chest wall mass, arising from the rib seen over upper lobe of the right lung. (c) Pulmonary nodules observed in lower lobe of the left lung.

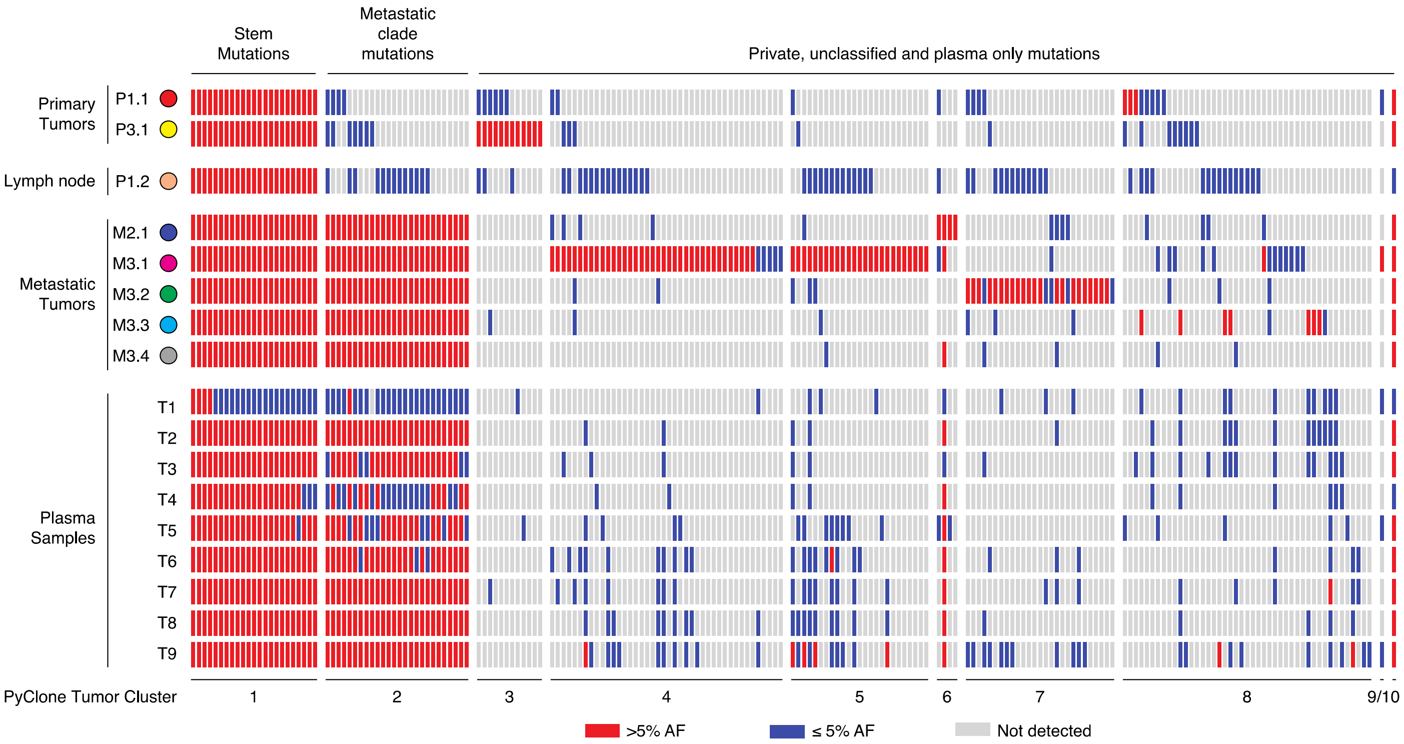

Supplementary Figure 8. Distribution of validated mutations using 5% allele fraction cut-off for high confidence.

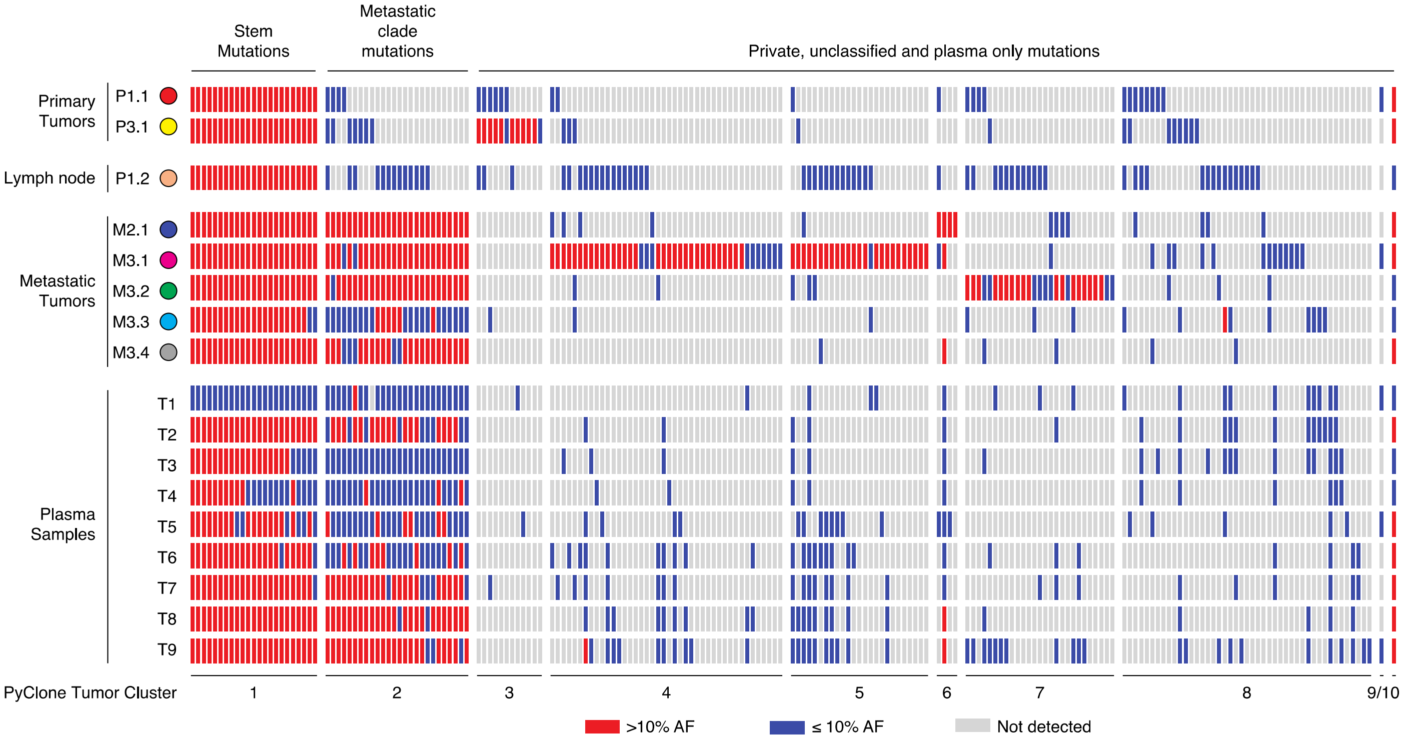

Supplementary Figure 9: Distribution of validated mutations using 10% allele fraction cut-off for high confidence.

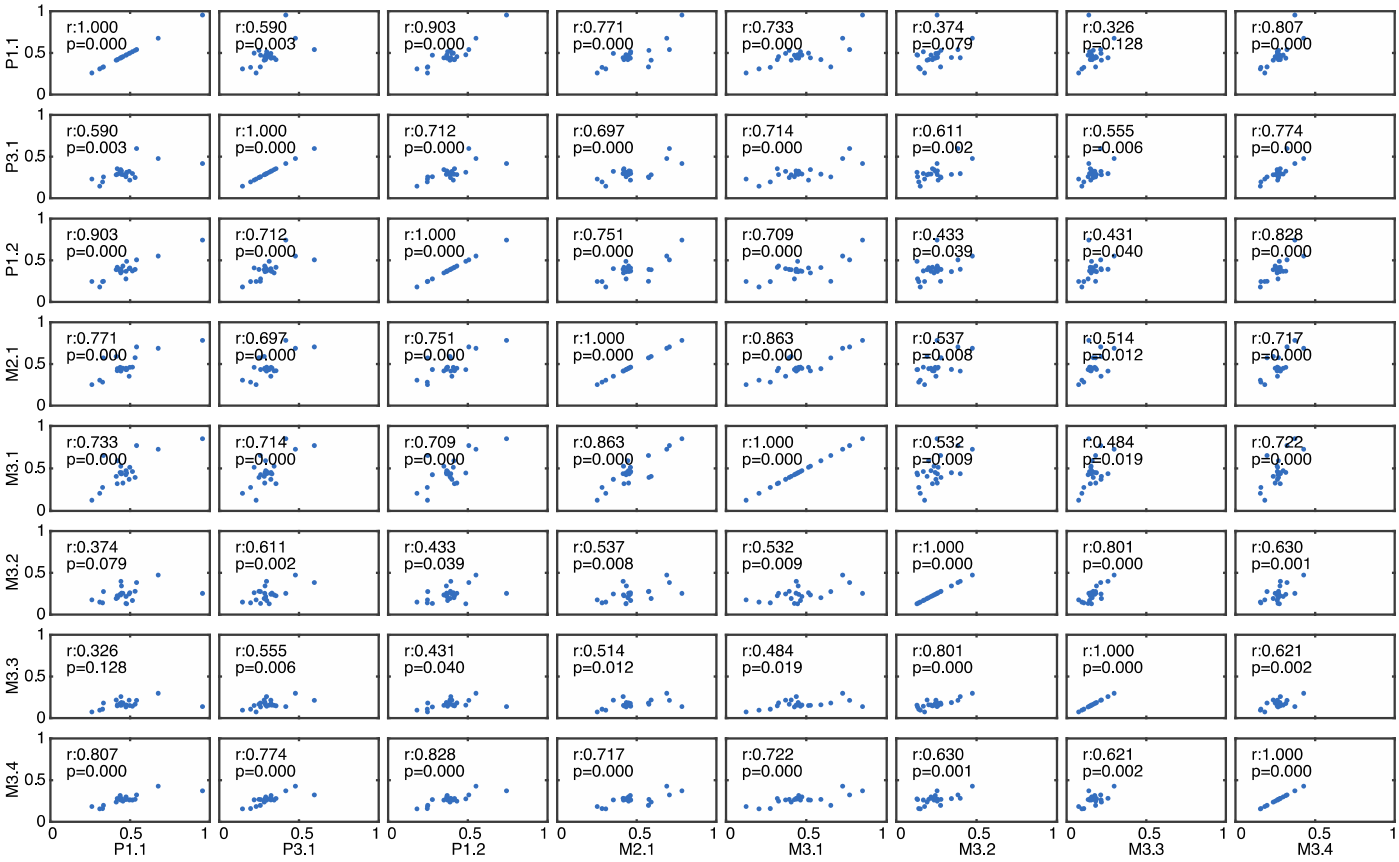

Supplementary Figure 10: Correlation of mutant allele fraction between tumor samples for stem mutations. r is Pearson's linear correlation co-efficient and p is the associated p-value from a two-tailed test.

a

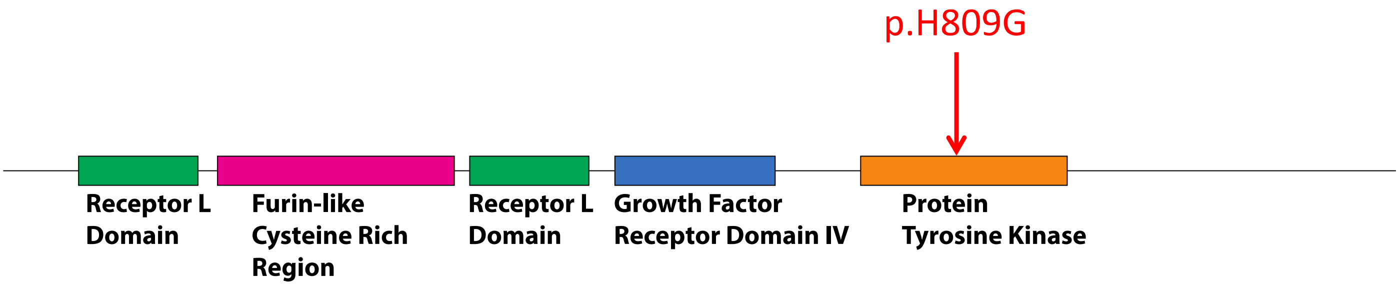

b

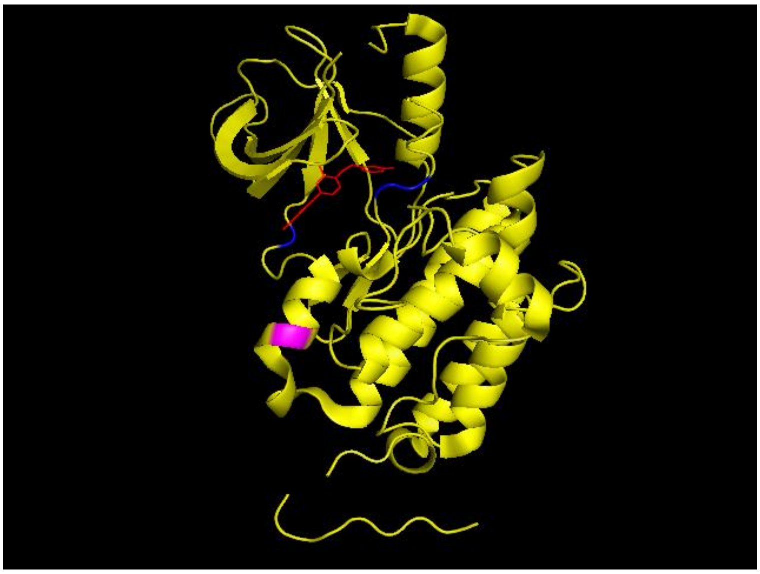

Supplementary Figure 11. Primary and crystal structure of the kinase domain of ERBB4 in the presence of lapatinib, highlighting the observed mutation. (a) Schematic structure of ERBB4 highlighting the location of the observed mutation p.H809G in the context of function domains. (b) Crystal structure shows residues of ERBB4 forming a hydrogen bond with lapatinib (shown in blue are amino acids 774, 836 and 837). In magenta is the site of the observed mutation p.H809G (adapted from Qui C et al.).[1]

Sample	Anatomic Site	Tumor fraction* (Mean AF of Stem Mutations)	Sample type
P1.1	Right Breast	20-30% (46.9%)	FFPE
P3.1	Right Breast	25% (30.7%)	FF
M2.1	Brain Mets	70% (47.1%)	FF
M3.1	Chest wall	90% (46.2%)	FF
M3.2	Liver	80% (24.5%)	FF
M3.3	Ovary	10% (16.9%)	FF
M3.4	Vertebrae	50-60% (26.8%)	FFPE

Supplementary Table 1. Tumor specimen cellularity assessed from H&E slides. *Of cells observed, the fraction judged to be cancerous. FF=Flash Frozen. FFPE=Formalin Fixed Paraffin Embedded

Days of follow up	Investigation	Indication	Summary of findings
5	CT Chest, Abdomen, Pelvis	Staging	Diffuse bony metastatic disease in axial skeleton<br>Bilateral pleural effusions but no focal pulmonary nodule<br>Multiple bilobar metastases in the liver<br>Increase in number but not size of right axillary lymph nodes<br>Retroperitoneal lymph node enlargement
26	Bone Scan	Staging	Diffuse bony metastatic disease
35	CT Head	Query of meningeal disease	No convincing evidence to suggest meningeal involvement<br>Small lytic lesion on right side of clivus
75	CT Chest, Abdomen, Pelvis	Assessment of Response (To Trastuzumab)	Right axillary nodes and retroperitoneal nodes almost resolved<br>Decrease in bilateral pleural effusions<br>Slight reduction in hepatic metastases<br>Sclerosis in ribs, vertebrae and pelvic bones
140	CT Chest, Abdomen, Pelvis	Assessment of Response	Persistent bilateral pleural effusions<br>Stable hepatic metastases
202	CT Chest, Abdomen, Pelvis	Assessment of Response	Persistent bilateral pleural effusions<br>Further response in hepatic metastases<br>Areas of lytic bony metastases with sclerotic change
258	CT Chest, Abdomen, Pelvis	Assessment of Response	No pleural effusions<br>No change in liver metastases since last examination<br>No lymphadenopathy
327	CT Chest, Abdomen, Pelvis	Assessment of Response	No changes seen since last examination
459	CT Chest, Abdomen, Pelvis	Assessment of Response	No changes seen since last examination
564	CT Head	Query of brain metastasis following seizures	Left frontal mass with contrast enhancement and surrounding edema<br>Midline shift<br>Small enhancing lesions in parietal and occipital regions
566	MRI Head	Surgical planning	Multiple lesions consistent with CT findings, largest in left frontal lobe
593	Bone Scan	Assessment of Response	Progression of widespread skeletal metastases
620	CT Chest, Abdomen, Pelvis	Assessment of Response	No pleural effusion or pulmonary nodules<br>Equivocal assessment of liver lesions<br>Small low-attenuation lesion noted in right kidney<br>Stable disease
700	CT Chest, Abdomen, Pelvis	Assessment of Response	No change on chest exam<br>Increase in size of liver lesions<br>New lesion noted in left ovary<br>Unremarkable kidneys<br>Progressive disease
804	CT Chest, Abdomen, Pelvis	Assessment of Response	No change on chest exam<br>Slight decrease in size of liver lesions<br>Left ovarian lesion unchanged<br>Stable disease
867	CT Chest, Abdomen, Pelvis	Assessment of Response	No lesions in the lung<br>Pleural lesion over right upper lobe<br>Slight reduction in hepatic lesions<br>Left ovarian lesion unchanged<br>Progression in skeletal metastatic disease
937	CT Chest,	Assessment of	No lesions in the lung

	Abdomen, Pelvis	Response	Increase in pleural lesion over right upper lobe<br>Marginal reduction in hepatic lesions<br>Left ovarian lesion unchanged<br>Stable disease
999	CT Chest, Abdomen, Pelvis	Assessment of Response	Interval increase in thoracic wall mass<br>No change in hepatic lesions<br>Stable disease
1077	CT Chest, Abdomen, Pelvis	Assessment of Response	Interval increase in right posterior chest wall mass<br>Three new pulmonary nodules in left lower lobe<br>Bilateral pleural effusions<br>No change in hepatic lesions<br>Progressive disease in chest, stable otherwise

Supplementary Table 2. Summary of Radiological Investigations and Findings.

	Exome				Targeted sequencing		
**Sample**	**Number of reads (millions)**	**Mean depth of coverage**	**Number of SNVs identified**	**Number of functional SNVs identified**	**Number of reads (millions)**	**Mean depth of coverage**	**Median depth of coverage**
N1 (matched normal)	327	26.1	NA	NA	1.99	4304	4452
P1.1 (breast, DCIS)	230	21.4	99	49	1.81	2477	2124
P3.1 (breast at autopsy)	134	74.2	122	60	1.19	2567	2352
P1.2 (lymph node)	-	-	-	-	1.19	1086	965
M2.1 (brain)	23	16.6	172	94	0.98	1891	1661
M3.1 (chest wall)	129	63.4	285	145	1.14	2631	2270
M3.2 (liver)	147	73.6	197	96	1.32	2682	2374
M3.3 (ovarian)	137	69.5	127	64	1.13	2299	2304
M3.4 (vertebral)	-	-	-	-	1.76	3169	2777
T1 (plasma)	115	77.3	40	20	1.44	2978	2594
T2 (plasma)	126	79.6	131	59	3.84	9051	8248
T3 (plasma)	-	-	-	-	1.15	2470	2337
T4 (plasma)	-	-	-	-	0.98	1805	1389
T5 (plasma)	-	-	-	-	1.13	319	288
T6 (plasma)	-	-	-	-	0.85	1853	1714
T7 (plasma)	-	-	-	-	1.04	2448	2012
T8 (plasma)	-	-	-	-	1.03	2391	2181
T9 (plasma)	227	139.6	172	75	1.25	2876	2534

Supplementary Table 3. Summary of sequencing data and single nucleotide variants identified.

Supplementary Reference

1. Qiu, C., *et al.* Mechanism of activation and inhibition of the HER4/ErbB4 kinase. *Structure* **16**, 460-467 (2008).
